# Effects of human insulin and insulin aspart preparations on levels of IGF-I, IGFBPs and IGF bioactivity in patients with type 1 diabetes

**DOI:** 10.1186/1472-6823-14-35

**Published:** 2014-04-11

**Authors:** Zhulin Ma, Jens Sandahl Christiansen, Torben Laursen, Chunsen Wu, Torsten Lauritzen, Tina Parkner, Jan Frystyk

**Affiliations:** 1 Medical Research Laboratory, Department of Clinical Medicine, Faculty of Health, Aarhus University, DK-8000 Aarhus C, Denmark; 2 Department of Endocrinology and Internal Medicine, Aarhus University Hospital, Nørrebrogade, DK-8000 Aarhus C, Denmark; 3 Department of Biomedicine - Pharmacology, Faculty of Health, Aarhus University, DK-8000 Aarhus C, Denmark; 4 Department of Public Health, Section for Epidemiology, Faculty of Health, Aarhus University, DK-8000 Aarhus C, Denmark; 5 Department of Public Health, Section of General Practice, Faculty of Health, Aarhus University, DK-8000 Aarhus C, Denmark; 6 Department of Clinical Biochemistry, Aarhus University Hospital, Nørrebrogade, DK-8000 Aarhus C, Denmark

**Keywords:** Insulin, Insulin analogue, IGF, IGFBP

## Abstract

**Background:**

Insulin aspart (IAsp) and its biphasic preparations BIAsp50 and BIAsp70 (containing 50% and 70% IAsp, respectively) have distinct glucose-lowering properties as compared to human insulin (HI). We investigated whether this affected the circulating IGF-system which depends on the hepatic insulin exposure.

**Methods:**

In a randomized, four-period crossover study, 19 patients with type 1 diabetes received identical doses (0.2 U/kg sc) of IAsp, BIAsp70, BIAsp50 and HI together with a standardized meal. Serum total IGF-I and IGFBP-1 to -3 were measured by immunoassays for nine hours post-prandially. Bioactive IGF was determined by an in-house, cell-based IGF-I receptor kinase activation (KIRA) assay.

**Results:**

Despite marked differences in peripheral insulin concentrations and plasma glucose, the four insulin preparations resulted in parallel decreases in IGFBP-1 levels during the first 3 hours, and parallel increases during the last part of the study (3–9 hours). Thus, only minor significances were seen. Insulin aspart and human insulin resulted in a lower area under the curve (AUC) during the first 3 hours as compared to BIAsp70 (*p* = 0.009), and overall, human insulin resulted in a lower IGFBP-1 AUC than BIAsp70 (*p* = 0.025). Nevertheless, responses and AUCs of bioactive IGF were similar for all four insulin preparations. Changes in levels of bioactive IGF were inversely correlated to those of IGFBP-1, increasing during the first 3 hours, whereafter levels declined (-0.83 ≤ r ≤ -0.30; all *p*-values <0.05).

Total IGF-I and IGFBP-3 remained stable during the 9 hours, whereas IGFBP-2 changed opposite of IGFBP-1, increasing after 3–4 hours whereafter levels gradually declined. The four insulin preparations resulted in similar profiles and AUCs of total IGF-I, IGFBP-2 and IGFBP-3.

**Conclusions:**

Despite distinct glucose-lowering properties, the tested insulin preparations had similar effects on IGF-I concentration and IGF bioactivity, IGFBP-2 and IGFBP-3 as compared to HI; only small differences in IGFBP-1 were seen and they did not affect bioactive IGF. Thus, insulin aspart containing preparation behaves as HI in regards to the circulating IGF-system. However, bioactive IGF appeared to be more sensitive to insulin exposure than total IGF-I. The physiological significance of this finding remains to be determined.

**Trial registration:**

NCT00888732

## Background

In patients with type 1 diabetes (T1D), insulinopenia in the portal circulation leads to alterations of the growth hormone - insulin-like growth factor I - insulin-like growth factor binding protein (GH - IGF-I - IGFBP) axis [1], including GH hypersecretion, reduced circulating levels of IGF-I and IGFBP-3, and elevated levels of IGFBP-1 and -2 [2-4]. Due to the insulin antagonizing effects of GH and the insulin sensitizing effects of IGF-I, these alterations are likely to have a negative effect on whole body insulin resistance [5,6] and they may also contribute to the development of long-term complications. Thus, in T1D one hypothesis has linked the augmented secretion of GH with the development of diabetic retinopathy [7] and nephropathy [8]. Supportive of this, intensified subcutaneous insulin therapy can improve metabolic control, return changes in the GH-IGF-axis towards normal and decrease progression of diabetes complications in patients with T1D [9]. However, it is worth to realize that not even excellent glycemic control can fully normalize the GH-IGF-I-IGFBP axis [2].

Insulin aspart, a rapid-acting insulin analogue, has been employed in intensified insulin therapy for many years. After subcutaneous injection, insulin aspart is absorbed faster and eliminated more quickly than human insulin, thus it has a more physiological insulin profile [10]. The pharmacokinetic profile of insulin aspart can be extended by combining soluble insulin aspart with protamine insulin aspart, but even this formulation has a faster onset of action than human insulin. Thus, a previous clinical study demonstrated that insulin aspart as well as mixtures of soluble and protamine insulin aspart (i.e. BIAsp70 and BIAsp50 which contains 70% and 50% rapid-acting insulin aspart and 30% and 50% protamine insulin aspart, respectively), are absorbed twice as fast as human insulin and associate with higher peak concentrations [11]. Based on these observations we hypothesized that the distinct peripheral insulin profiles obtained with the abovementioned insulin preparations could translate into distinct hepatic exposures to insulin and in this way affect the liver production of IGF-I and IGFBPs differently. However, this assumption has not been tested under controlled and standardized circumstances.

The aim of the present trial was to compare the serum profiles of IGF-I and the IGFBPs after a subcutaneous injection with equal doses of insulin aspart, BIAsp70, BIAsp50 or human insulin. In particularly, their effects on the insulin-regulated IGFBP-1 had our interest, as differences in IGFBP-1 may lead to secondary changes in bioactive IGF.

## Methods

This open-label, randomized, four-period crossover study was approved by the central Denmark region committees on health research ethics and the Danish Medical Agency. The study was carried out according to the Declaration of Helsinki and the principles of Good Clinical Practice. Written informed consent was obtained from all the participants before any study related activities. Findings related to the pharmacokinetic and pharmacodynamic profiles of human insulin vs. the three different insulin aspart preparations have recently been published elsewhere [11].

### Patients

Patients of both genders with T1D, aged ≥ 18 years, with HbA1c of 53–108 mmol/mol (7-12%) and body mass index (BMI) of 18–35 kg/m^2^ were recruited. Patients had to have the clinical diagnosis of diabetes before the age of 40 years and to be treated with any insulin regimen for ≥ 12 months at a daily insulin dose ≥ 0.4 U/kg. Exclusion criteria included patients who had allergy to investigational insulin, recurrent major hypoglycemia (defined as >2 severe hypoglycemic episodes within the last 12 months), acute myocardial infarct < 12 months or severe heart insufficiency, abnormal liver and kidney function (estimated by routine laboratory biochemical testing), pregnancy or breast-feeding. Some patients received other prescribed drugs than insulin; however, this medication remained unaltered during the entire study.

### Procedures

The study comprised four study visits (at least one week apart), where insulin aspart, BIAsp70, BIAsp50 or human insulin were administrated in random order. By choosing a crossover design where each patient served as his or her own control, we aimed to reduce the influence of inter-individual differences in regards to for instance insulin sensitivity. Patients reported to the clinical research unit at 9:00 p.m. on the night prior to the profile day. They were thoroughly instructed to inject the last dose of basal insulin at least 24 hours before administration of the study insulin. During the overnight fast, intravenous infusions of isotonic glucose and insulin were administered to maintain blood glucose values between 5 and 8 mM and by this, to attain a comparable baseline glucose level prior to study insulin injection. Infusions were commenced at 10:00 p.m. and ended at 8:00 a.m. Soluble human insulin (Actrapid^®^, Novo Nordisk) was infused during the night prior to the morning injection of insulin aspart preparations. Conversely, insulin aspart was infused before the morning injection of human insulin. Blood glucose was measured every 30 min over night. Before the insulin injection, the glucose values had to be within the defined range for at least 30 min. In the morning of the profile day, a single dose (0.2 U/kg) of either insulin aspart, BIAsp70, BIAsp50 or human insulin (Actrapid^®^, Novo Nordisk) was injected subcutaneously at the abdomen. Insulin aspart preparations were injected immediately before the meal, which started at 8 a.m., whereas human insulin was injected 30 min before the meal. The standardized meal contained 3360 kJ fat, carbohydrate and protein and was finished within 15 min. From 8:00 a.m. (baseline), blood samples were drawn hourly for determination of serum concentrations of IGFBP-1 and IGFBP-2, while total IGF-I, bioactive IGF and IGFBP-3 were measured at 0, 3, 6, and 9 hours.

Blood glucose was measured every hour with the plasma glucose meter (Ascensia Contour, Bayer) for safety assessment and more often if needed. Patients were treated with oral glucose (equal to 40 g glucose) or intravenous injection of 10% glucose if the blood glucose measurement was below 3.1 mM. Study procedures were discontinued when the patient’s glucose measurement exceeded 16.0 mM.

### Laboratory assessment

All the variables were analyzed in serum. Samples were stored at -80°C until analysis.

Total IGF-I and IGFBP-3 levels were determined by commercial chemiluminescence immunoassays on the IDS-iSYS multi-discipline automated analyzer (Immunodiagnostic Systems Nordic SA, Denmark) according to the manufacturer’s protocol, as recently published [12,13]. Limits of detection for total IGF-I and IGFBP-3 were 4.4 μg/l and 50 μg/l respectively.

IGFBP-1 was determined by an in-house time-resolved immunofluorometric assay (TR-IFMA) with slight modifications as recently described [14]. IGFBP-2 was determined by a validated in-house time-resolved immunofluorometric assay (TR-IFMA) as previously described [15]. The IGFBP-1 and -2 assays have intra- and inter-assay CVs averaging <5% and <12%.

Bioactive IGF was determined by an IGF-I kinase receptor activation (KIRA) assay based on human IGF-I receptor gene-transfected embryonic renal cell as described by Chen *et al.*[16] with slight modifications [14]. In brief, transfected cells were stimulated for 15 min at 37°C with either IGF-I standards (WHO 02/254) or diluted serum. After cells were aspirated and the cells lysed, the crude cell lysates were transferred to a TR-IFMA assay to detect the concentration of phosphorylated IGF-I receptors. The KIRA assay also detects IGF-II and pro-IGF-II activation of the IGF-IR with a cross-reactivity of 12% and 2%, respectively. Although the majority of the KIRA assay signal obtained in serum without any doubt originates from an IGF-I induced activation of the IGF-IR, IGF-II is likely to participate and accordingly, the output of the KIRA assay has been named “IGF bioactivity”. By contrast, the cross reactivity of proinsulin, insulin and insulin analogues were less than 1%. The KIRA assay has a detection limit < 0.1 μg/l, and intra- and inter-assay CVs of <7% and <15%, respectively.

### Statistical analysis

A power analysis showed that 19 completed subjects would be required to yield a statistical power of 90% to detect an absolute difference of 10 μg/l between the minimum concentrations of IGFBP-1. Thus, to allow dropouts we invited 24 subjects to participate. Statistical analyses were performed using IBM SPSS Statistics version 20.0 (IBM Corp., Somers, NY, USA). Komogorov-Smirnov test was used to test for normal distribution. Natural logarithmic transformation was performed to improve the distribution of raw data. Area under the concentration-time curve (AUC) was calculated by the trapezoidal rule for the time intervals 0–3 hours (AUC_0–3_), 3–6 hours (AUC_3–6_), 6–9 hours (AUC_6–9_) and for all 9 hours (AUC_0–9_). The differences in Cmax, Cmin, tmax, tmin and AUCs were determined using analysis of variance (ANOVA) with treatment as a fixed factor and patient as a random factor. The baselines levels were used as co-variables in the ANOVA analyses for the AUC_0–3_ and AUC_0–9_. If a patient discontinued earlier than planned, ANOVA tests were only performed for his/her completed periods. If the ANOVA was significant, the difference between treatments was examined statistically by using the Bonferroni method. Analysis of time-related effects among groups was performed using repeated measures ANOVA. Linear correlations were used to assess relationships between measured variables. *P* values <0.05 were considered statistically significant.

## Results

Nineteen patients (15 men and 4 women), aged 40.7 (22–63) years (mean and range), with a BMI of 25.5 (20.6-30.3) kg/m^2^, a duration of T1D of 20.9 (3–47) years and a present HbA1c of 66 (53–84) mmol/mol [8.2 (7.0-9.8)%] underwent all four study visits. The profile day was ended after six hours of insulin injection for three patients with the insulin aspart treatment and for one patient with human insulin, because their glucose levels exceeded 16.0 mM as defined by study protocol. The rest of the patients completed the nine hours experimental period of all four study visits.

### Insulin and glucose

**Figure 1:**
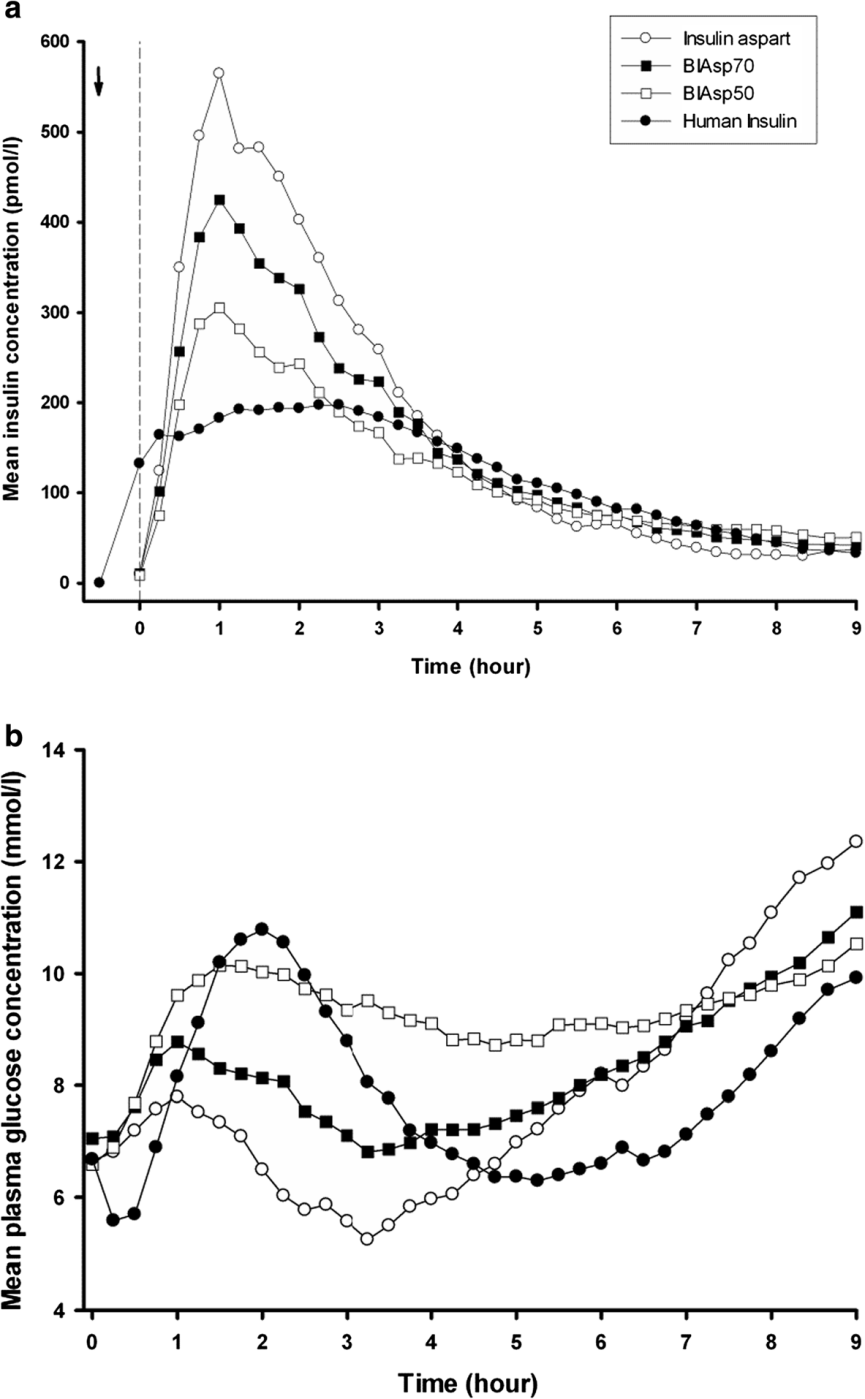
**Insulin and glucose profiles.** Serum insulin concentration **(a)** and plasma glucose concentration **(b)** during 9-hour treatment with insulin aspart (open circles), BIAsp70 (black rectangles), BIAsp50 (open rectangles) and human insulin (black circles). The arrow indicates human insulin injection time, and the vertical dotted line indicates the time of meal. Serum insulin and plasma glucose profiles are illustrated as mean levels (reprinted with permission from Diabetes Technology & Therapeutics).

Figure 1a and b illustrate the insulin and glucose profiles during nine hours period, as recently published [11]. In brief, results showed that the insulin and glucose profiles following sc injection of human insulin differed significantly from those of the insulin aspart preparations. BIAsp50 demonstrated the greatest similarity to human insulin as regards pharmacokinetic and pharmacodynamic profiles [11].

### Baseline IGF levels

At baseline, serum concentrations of total IGF-I, bioactive IGF and IGFBP-1 to -3 were comparable among the four treatment days (Table 1).

**Table 1:** Results of IGF parameters

**Parameter**	**Unit**	**Insulin aspart (a)**	**BIAsp70 (b)**	**BIAsp50 (c)**	**Human insulin (d)**	***p***
**Total IGF-I**						
Baseline	μg/l	113 (109–117)	121 (116–125)	117 (113–121)	115 (111–120)	NS
Cmax	μg/l	124 (121–126)	124 (122–126)	122 (120–125)	122 (119–124)	NS
Cmin	μg/l	113 (111–114)	113 (111–115)	112 (110–114)	113 (111–115)	NS
AUC0-3 h	μg*h/l	353 (349–357)	355 (351–359)	353 (349–357)	353 (350–357)	NS
AUC3-6 h	μg*h/l	349 (337–362)	370 (358–383)	356 (344–369)	352 (340–365)	NS
AUC6-9 h	μg*h/l	346 (333–361)	364 (352–378)	351 (339–364)	349 (336–362)	NS
AUC0-9 h	μg*h/l	1071 (1053–1090)	1071 (1055–1088)	1059 (1043–1074)	1064 (1048–1081)	NS
**Bioactive IGF**						
Baseline	μg/l	0.60 (0.54-0.67)	0.62 (0.56-0.69)	0.68 (0.61-0.76)	0.58 (0.52-0.65)	NS
Cmax	μg/l	0.80 (0.76-0.84)	0.79 (0.75-0.83)	0.79 (0.75-0.84)	0.82 (0.78-0.87)	NS
Cmin	μg/l	0.46 (0.43-0.50)	0.50 (0.47-0.54)	0.52 (0.49-0.56)	0.52 (0.48-0.56)	NS
AUC0-3 h	μg*h/l	2.02 (1.96-2.09)	1.96 (1.90-2.02)	2.04 (1.98-2.11)	2.08 (2.02-2.15)	.051
AUC3-6 h	μg*h/l	2.16 (2.02-2.31)	2.11 (1.98-2.25)	2.26 (2.11-2.41)	2.27 (2.13-2.43)	NS
AUC6-9 h	μg*h/l	1.82 (1.68-1.98)	1.90 (1.76-2.05)	1.99 (1.85-2.15)	1.98 (1.83-2.14)	NS
AUC0-9 h	μg*h/l	6.11 (5.80-6.45)	5.98 (5.71-6.27)	6.18 (5.89-6.49)	6.43 (6.12-6.75)	NS
**IGFBP-1**						
Baseline	μg/l	130 (107–159)	140 (115–171)	116 (95–141)	144 (118–176)	NS
Cmin	μg/l	14 (11–18)^c^	21 (16–27)	26 (20–34)^a^	22 (17–29)	.009
Tmin	min	259 (234–284)	256 (231–280)	231 (206–255)	268 (244–293)	NS
AUC0-3 h	μg*h/l	262 (233–294)^c^	319 (284–359)	351 (312–396)^a,d^	256 (228–288)^c^	.001
AUC3-6 h	μg*h/l	112 (81–155)	132 (96–183)	164 (119–227)^d^	83 (60–114)^c^	.029
AUC6-9 h	μg*h/l	662 (496–884)	577 (445–747)	455 (351–589)	390 (298–510)	NS
AUC0-9 h	μg*h/l	1092 (906–1317)	1084 (917–1282)^d^	1032 (871–1224)	779 (654–927)^b^	.025
**IGFBP-2**						
Baseline	μg/l	342 (312–375)	356 (324–390)	356 (325–391)	380 (347–417)	NS
Cmax 0-6 h	μg/l	477 (459–497)	473 (455–493)	462 (444–480)	465 (447–485)	NS
Tmax 0-6 h	min	215 (185–245)	215 (185–245)	218 (188–248)	205 (175–235)	NS
AUC0-3 h	μg*h/l	1225 (1191–1261)	1224 (1189–1259)	1184 (1151–1219)	1212 (1177–1247)	NS
AUC3-6 h	μg*h/l	1278 (1170–1395)	1303 (1194–1423)	1263 (1157–1378)	1363 (1249–1488)	NS
AUC6-9 h	μg*h/l	1127 (1015–1251)	1261 (1149–1385)	1221 (1112–1340)	1247 (1132–1374)	NS
AUC0-9 h	μg*h/l	3717 (3548–3892)	3758 (3609–3916)	3640 (3495–3792)	3651 (3498–3813)	NS
**IGFBP-3**						
Baseline	mg/l	3.04 (2.97-3.11)	3.12 (3.05-3.19)	3.05 (2.97-3.12)	3.01 (2.94-3.08)	NS
Cmax	mg/l	3.30 (3.23-3.37)	3.32 (3.25-3.39)	3.30 (3.24-3.37)	3.25 (3.18-3.32)	NS
Cmin	mg/l	2.98 (2.94-3.02)	3.00 (2.96-3.04)	2.98 (2.94-3.02)	2.99 (2.95-3.03)	NS
AUC0-3 h	mg*h/l	9.23 (9.12-9.34)	9.22 (9.11-9.33)	9.23 (9.12-9.34)	9.20 (9.09-9.31)	NS
AUC3-6 h	mg*h/l	9.40 (9.16-9.66)	9.67 (9.42-9.93)	9.44 (9.19-9.69)	9.26 (9.02-9.51)	NS
AUC6-9 h	mg*h/l	9.45 (9.19-9.72)	9.87 (9.63-10.12)	9.60 (9.36-9.84)	9.46 (9.22-9.71)	NS
AUC0-9 h	mg*h/l	28.19 (27.71-28.68)	28.50 (28.05-28.94)	28.33 (27.90-28.77)	28.16 (27.71-28.61)	NS

The results for Cmax, Cmin, Tmax, Tmin and AUCs are expressed as geometric mean (range) drawn from ANOVA. The outcomes were controlled for baseline levels (t = 0) if necessary, ^a^p < 0.05 vs insulin aspart, ^b^p < 0.05 vs BIAsp70, ^c^p < 0.05 vs BIAsp50, ^d^p < 0.05 vs human insulin.

### Changes in IGF levels during the nine hours of study

**Figure 2:**
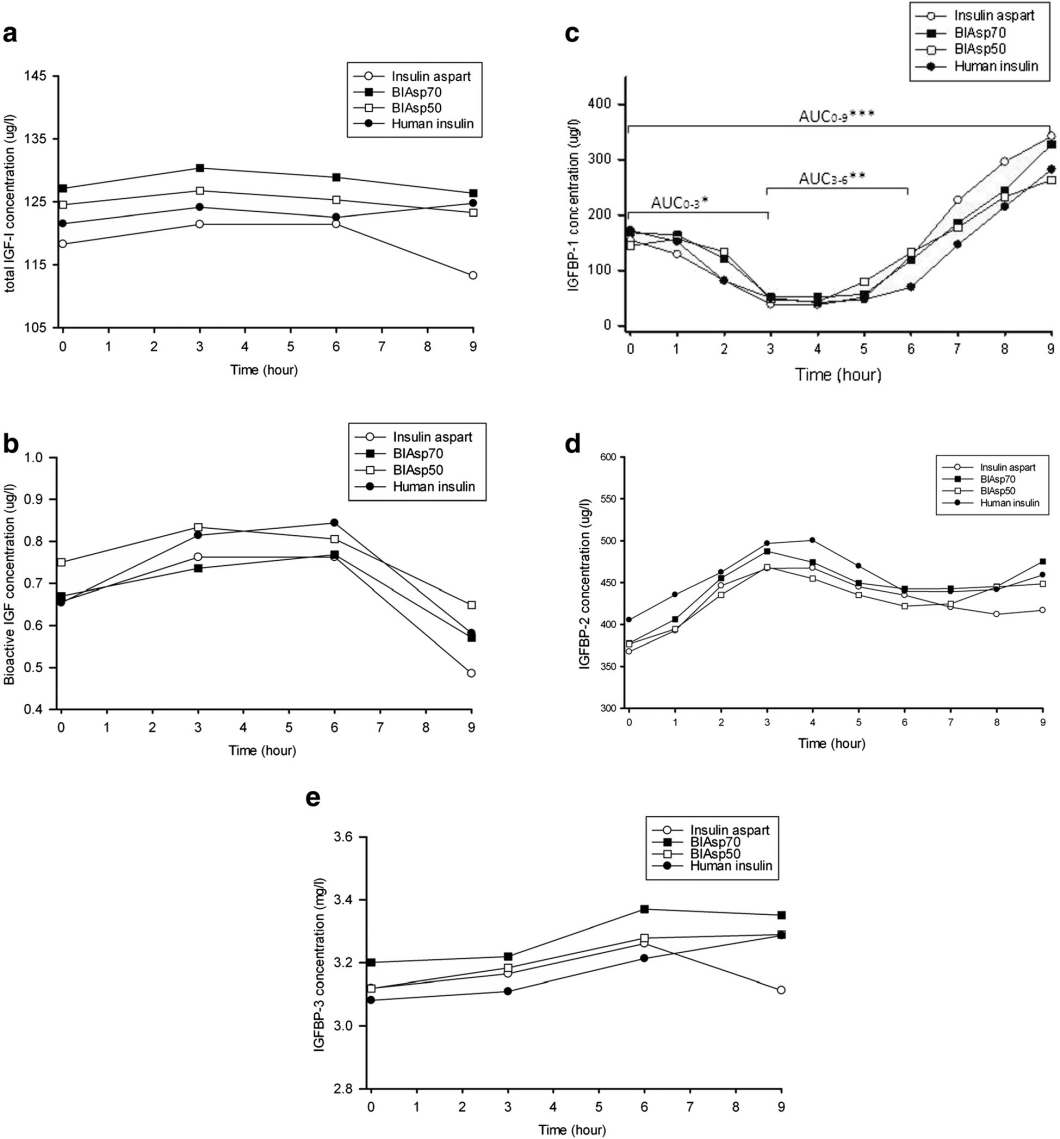
**IGF parameters profiles. (a)** Serum total IGF-I concentration, **(b)** bioactive IGF concentration, **(c)** IGFBP-1 concentration, *P < 0.01 as BIAsp50 compared with insulin aspart and human insulin for AUC_0–3_; **P < 0.05, as BIAsp50 compared with human insulin for AUC_3–6_; ***P < 0.05, as BIAsp70 compared with human insulin for AUC_0–9._**(d)** IGFBP-2 levels and **(e)** IGFBP-3 levels during 9-hour treatment with insulin aspart (open circles), BIAsp70 (black rectangles), BIAsp50 (open rectangles) and human insulin (black circles). For clarity, serum concentrations are illustrated as mean levels only.

None of the investigational insulin preparations changed serum total IGF-I levels from baseline (Figure 2a). Similarly, maximum and minimum concentrations of total IGF-I were similar for insulin aspart preparations and human insulin. There were no significant differences in the AUCs of total IGF-I in any time interval during the study (Table 1).

**Figure 3:**
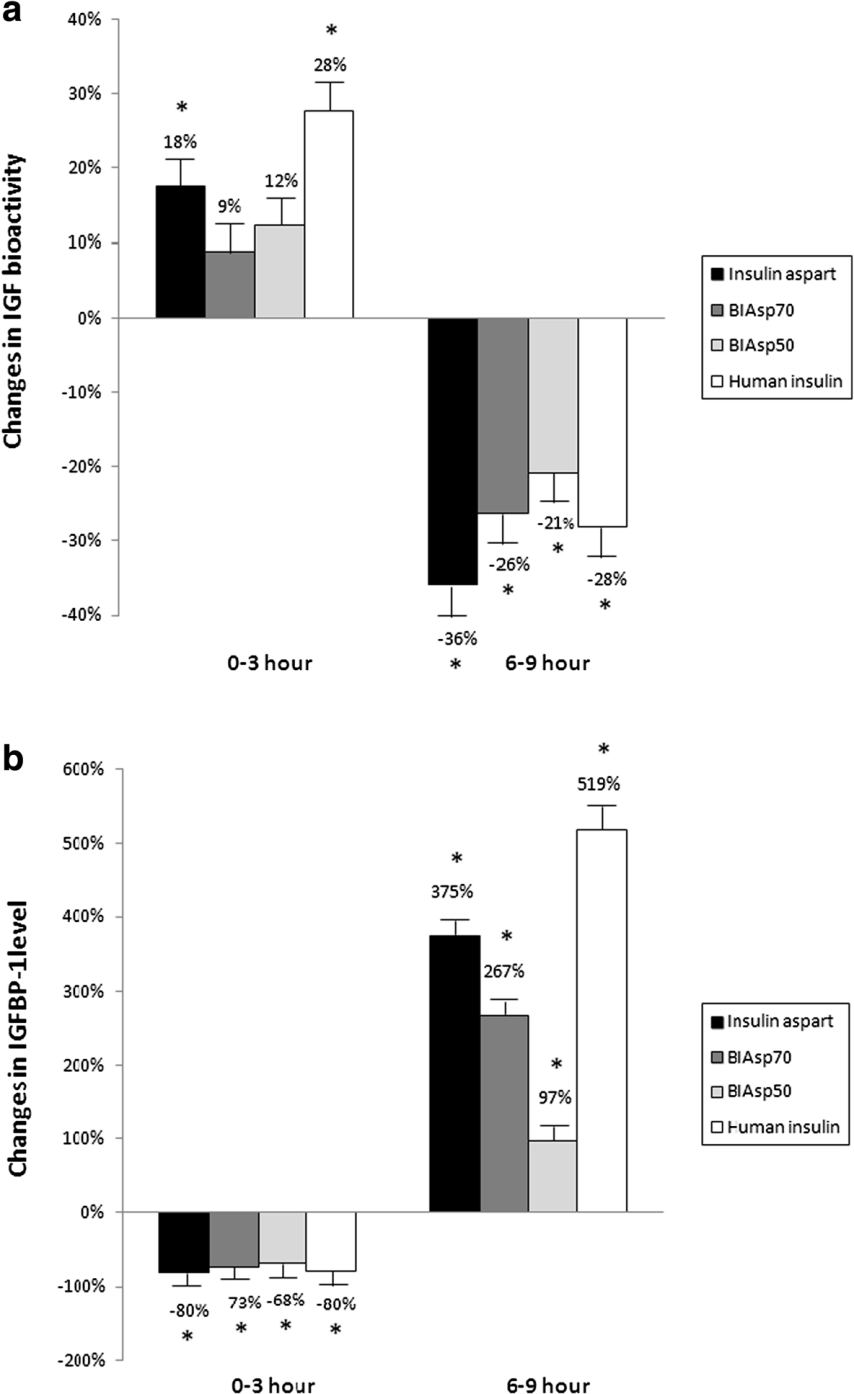
**Changes in IGF bioactivity and IGFBP-1.** IGF bioactivity **(a)** and IGFBP-1**(b)** levels during 0–3 hours and 6–9 hours. Asterisks indicate that significant change was found in comparison with previous level (*p* < 0.05).

For all four insulin preparations, serum IGF bioactivity increased during the first three hours. As compared to baseline levels, these increases were significant following insulin aspart (+18%) and human insulin (+28%), and insignificant for BIAsp70 (+9%) and BIAsp50 (+12%) (Figures 2b and 3a). By contrast, bioactive IGF levels decreased significantly (all *p*-values <0.05) during the last three hours, irrespective of study insulin (Figures 2b and 3a). In regards to Cmax, Cmin and the AUCs, no statistical significant differences in bioactive IGF were found between the four insulin preparations (Table 1).

**Figure 4:**
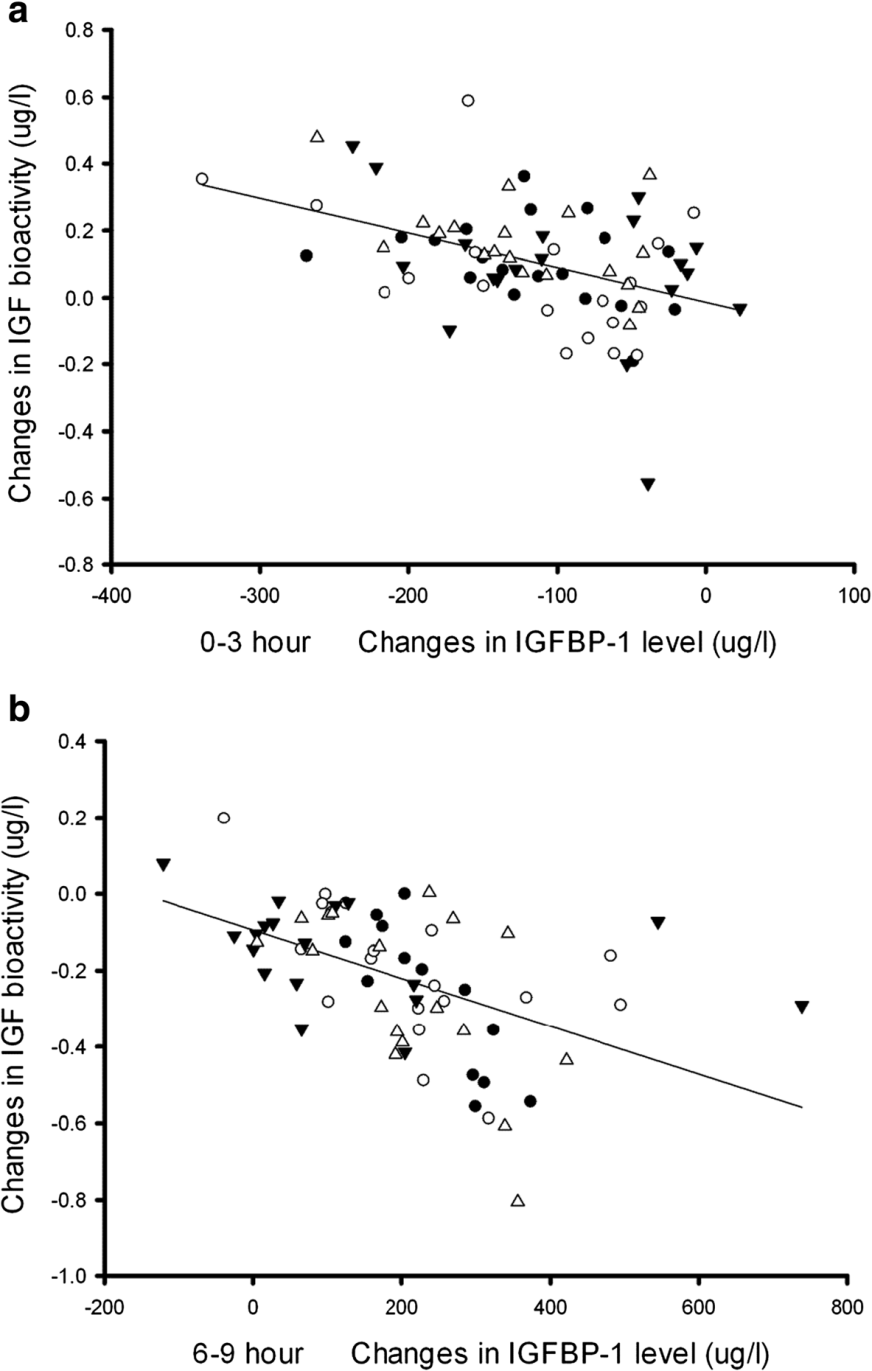
**Linear regression between the changes of IGF bioactivity and IGFBP-1 level.** 0–3 hours **(a)** and 6–9 hours **(b)**. The *symbols* represent patients with insulin aspart (black circles), BIAsp70 (open circles), BIAsp 50 (black triangles) and human insulin (open triangles). For all four insulin preparations a significant inverse correlation with IGFBP-1 was observed, being r = - 0.30 for insulin aspart, r = - 0.47 for BIAsp70, r = - 0.40 for BIAsp50, and r = - 0.52 for human insulin group during 0–3 hours **(a)**, while r = - 0.83 for insulin aspart, r = - 0.57 for BIAsp70, r = - 0.40 for BIAsp50, and r = - 0.58 for human insulin group during 6–9 hours **(b)**; all *p*-values <0.05. For clarity the reference line illustrates the trend of data in four insulin groups.

For all four insulin preparations, linear regression analysis showed that changes in IGFBP-1 correlated inversely with changes in IGF bioactivity (Figures 3 and 4). Three hours after the administration of study insulin, serum IGFBP-1 reached nadir, whereafter levels gradually increased to peak values at the end of the study (all *p*-values <0.05). Although the four different insulin preparations resulted in parallel changes in serum IGFBP-1, a detailed analysis revealed minor but significant differences when comparing the AUCs (for details please refer to Table 1). In general, insulin aspart preparations with higher proportions of the rapid-acting aspart induced lower IGFBP-1 AUCs during the first six hours and higher AUCs during the last three hours (Table 1).

Serum IGFBP-2 increased in the early phase of the profile day whereafter levels declined towards baseline values. Neither peak levels nor AUCs differed when comparing the different insulin preparations (Table 1).

Serum IGFBP-3 remained unchanged during all four profile days. Although IGFBP-3 appeared to display differences during the last three hours, these differences were insignificant (Figure 2e and Table 1).

## Discussion

We have previously demonstrated that equal doses of human insulin, insulin aspart and the two biphasic aspart preparations BIAsp50 and BIAsp70 have distinct pharmacokinetic and -dynamic properties in patients with T1D [11] and this finding prompted us to investigate their effect on the circulating IGF system, which is known to respond to insulin. However, as scrutinized by the present study, the distinct pharmacokinetic properties of the tested insulin preparations only affected IGFBP-1 and these changes were minor and hardly of any major physiological significance. Supportive of this interpretation, levels of bioactive IGF responded similarly to the four insulin preparation and the same was true for total IGF-I, IGFBP-2 and IGFBP-3. On the other hand, our study clearly demonstrates bioactive IGF to be more sensitive to short-term changes in insulin exposure than total IGF-I. This observation highlights the important regulatory role of insulin (human as well as analogues) on the IGF system.

IGFBP-1 is the only component of the IGF system that is directly regulated by insulin, which inhibits the hepatic synthesis of IGFBP-1 at the transcriptional level [17-19]. As the liver is considered to be the main source of IGFBP-1, its serum levels can be used to estimate the hepatic exposure to insulin [17] as well as the hepatic sensitivity to insulin [20]. As expected, serum IGFBP-1 declined after sc administration of insulin, irrespective of type of insulin. However, some differences were indeed observed when comparing the four preparations. Overall, during the 9 hours of study human insulin resulted in the lowest IGFBP-1 concentrations. Furthermore, pure aspart differed from the two biphasic aspart preparations when comparing early and late effects, but overall the three aspart-containing preparations yielded similar AUCs. Whether these minor differences have any physiological importance remain uncertain. However, IGFBP-1 is an important regulator of free IGF-I [21] as well as bioactive IGF [22], at least when measured *in vitro*, and as we observed no differences in the response of bioactive IGF to the four insulin preparations, we speculate that the observed statistical differences in IGFBP-1 has little, if any biological significance. Thus, we conclude that despite clearly distinct pharmacokinetic and -dynamic profiles of the four insulin preparations, this did not translate into clearly distinct IGFBP-1 profiles. As we have no reasons to believe that the insulin sensitivity of the participants changed during the study period, this indicates that the four tested insulin preparations resulted in comparable hepatic insulin exposures and this we believe is valuable clinical information. Importantly, our observation is in agreement with a previous publication by Hedman *et al.*, who compared 6 weeks of constant subcutaneous infusion of insulin lispro vs. human insulin [23]. These authors observed marked postprandial differences in blood glucose and plasma insulin, whereas the postprandial excursions of serum IGFBP-1 did not differ between the two insulin preparations. Thus, it seems fair to conclude that the glycemic response to rapid-acting insulin preparations cannot be used to predict the response of the IGF-system.

Circulating IGFBP-1 only constitute a minor fraction of the six IGFBPs [18] but it is, nevertheless, believed to act as an important short-term regulator of IGF actions *in vivo*[24]. Thus, it is generally believed that reductions in circulating IGFBP-1 result in a diminution of IGF binding capacity and consequently increases free and subsequently bioactive IGF [5,24]. With the cell based IGF-IR bioassay at hand we are able to determine the ability of serum to activate the IGF-IR *in vitro*[16]. Our findings demonstrated a strong interaction between IGF bioactivity and circulating IGFBP-1 levels. During the first three hours, a reduction in IGFBP-1 levels was accompanied by an increase in IGF bioactivity. Conversely, at the end of the profile day (6–9 h), the marked increases in IGFBP-1 associated with a large decline in IGF bioactivity. These findings illustrate the intimate relationship between insulin and the IGF system and suggests that in patients with T1D the actions of the IGF system may be more fluctuating and dependent on insulin administration than what is suggested from measurements of total IGF-I. However, we acknowledge that the clinical relevance of our finding needs to be clarified.

Unlike IGFBP-1 and bioactive IGF, total IGF-I and IGFBP-3 remained constant during the nine hours of study and no significant differences in total IGF-I outcomes could be detected among the four insulin preparations. This finding was, however, not surprising as the majority of circulating IGF-I is bound in ternary complexes composed of IGFBP-3 and the acid-labile subunit (ALS). These complexes are primarily dependent on GH rather than insulin and furthermore characterized by a circulating half-life of 12–15 hours [25,26].

Studies in humans and in experimental animal models have shown that IGFBP-2 is a key factor in the insulin-IGF cross-talk and also linked to insulin resistance [27-30]. Our study demonstrated for the first time that serum IGFBP-2 fluctuates after a meal-time insulin injection in patients with T1D, hereby supporting its metabolic relationship. As for IGFBP-1, the four insulin preparations resulted in virtually similar changes in IGFBP-2 levels, regardless of glycemic control. Whether the increase in IGFBP-2 serves to counteract the impact of IGFBP-1 on bioactive IGF remains unknown, but this is most likely, as IGFBP-2 has been demonstrated to correlate inversely with levels of bioactive IGF following a hyperinsulinemic clamp [28].

The present study has some limitations that should be taken into consideration for the interpretation of the results. Firstly, we compared peripheral levels of the different insulin preparations and they could differ from levels within the portal vein system. Secondly, we had a priori decided to withdraw patients when their plasma glucose exceeded 16 mM. This decision reduced the number of samples collected at six and nine hours and it may have contributed to underestimate any late occurring differences between the four insulin preparations. Thirdly, we have to acknowledge that the calculated dose of insulin was insufficient to maintain postprandial blood glucose levels within normal range. Thus, we cannot fully preclude that the use of higher insulin doses may have enhanced our ability to detect significant differences in the response of the IGF-system.

Molecular modification of human insulin, resulting in insulin analogues, aims to improve pharmacokinetic and -dynamic properties, but at the same time this may increase IGF-IR binding affinity, hereby raising clinical safety concerns. In this context, studies have demonstrated that insulin aspart resembles human insulin in its IGF-IR binding affinity and mitogenic potency [31]. In fact, the capacity of insulin aspart to cross-react with the IGF-IR *in vitro* was slightly lower than that of human insulin [16]. These findings were supported by the present clinical study demonstrating no increased IGF-IR activation *in vitro* following treatment with insulin aspart containing preparations. Furthermore, all three insulin aspart preparations had close to identical effects on the circulating IGF system as compared to human insulin, the only exception being slight differences in IGFBP-1, which however, were expected and in accordance with the pharmacokinetic properties of the tested insulins. Thus, in regards to short term effects on the circulating IGF system, our data yield no reason to concern when using insulin aspart containing preparations.

## Conclusion

The present study demonstrated that despite distinct pharmacokinetic and -dynamic properties, insulin aspart preparations had similar effects on IGF-I concentration and IGF bioactivity as well as on levels of IGFBP-2 and IGFBP-3 as compared to those of human insulin. Differences were observed in regards to the very insulin sensitive protein IGFBP-1, but the magnitude of these differences were small and hardly on any major biological significance as they did not impact bioactive IGF. On the other hand, bioactive IGF appeared to be more sensitive to insulin exposure than total IGF-I, and hence our study suggests that the activity of the IGF system may be relatively dynamic in patients with T1D, being dependent on the prevailing insulin levels. However, the clinical and pathophysiological importance of such insulin-related fluctuations in IGF bioactivity remains to be clarified.

## Abbreviations

ANOVA: Analysis of variance; AUC: Area under the concentration-time curve; BIAsp: Biphasic insulin aspart; Cmax: Maximal concentration; Cmin: Minimal concentration; GH: Growth hormone; IGF: Insulin-like growth factor; IGFBP: Insulin-like growth factor binding protein; IGF-IR: IGF-I receptor; Tmax: Time to maximal concentration; Tmin: Time to minimal concentration.

## Competing interest

The study was supported by an unrestricted grant from Novo Nordisk A/S. Zhulin Ma is the recipient of unrestricted grants for research from Novo Nordisk. Jens Sandahl Christiansen is the member of speaker’s bureau and Advisory Boards and recipient of unrestricted research grants from Novo Nordisk. Torsten Lauritzen have received unrestricted grants for the ADDITION study (screening and intensive treatment of type 2 diabetes in primary care) from public foundations and the Medical Industry: Novo Nordisk AS, Novo Nordisk Scandinavia AB, ASTRA Denmark, Pfizer Denmark, GlaxoSmithKline Pharma Denmark, SERVIER Denmark A/S and HemoCue Denmark A/S. Torsten Lauritzen has held 3 lectures for the medical industry within the past 2 years. Torsten Lauritzen hold shares in Novo Nordisk. The other authors have nothing to disclose.

## Authors’ contributions

ZM carried out the clinical study, performed the statistical analysis and wrote the manuscript. JSC, TL and JF conceived of the study, participated in its design and coordination and helped to draft the manuscript. TL and TP helped to interpret results and revised the manuscript. CSW participated in the design of data management plan and performed the partial statistical analysis. All authors read and approved the final manuscript.

## Pre-publication history

The pre-publication history for this paper can be accessed here:

http://www.biomedcentral.com/1472-6823/14/35/prepub
